# Psychosocial Factors in Help-Seeking Among Older Adults During the COVID-19 Pandemic: A Qualitative Analysis of Calls to India’s National Psychosocial Support and Mental Health Services Helpline

**DOI:** 10.7759/cureus.105375

**Published:** 2026-03-17

**Authors:** Allen D Christopher, Jayakumar Christy, Bangalore Roopesh, Sanjeev K Manikappa, Marimuthu Palaniyappan

**Affiliations:** 1 Psychosocial Support in Disaster Management, National Institute of Mental Health and Neurosciences, Bengaluru, IND; 2 Clinical Psychology, National Institute of Mental Health and Neurosciences, Bengaluru, IND; 3 Biostatistics, National Institute of Mental Health and Neurosciences, Bengaluru, IND

**Keywords:** covid-19 pandemic, help-seeking behavior, mental health helpline, older adults, psychosocial support

## Abstract

Background: Older adults faced significant psychosocial challenges during the COVID-19 pandemic, yet evidence on help-seeking behavior in low- and middle-income countries remains limited. This study aimed to identify the psychosocial factors prompting help-seeking among older Indian adults and family members who contacted the National Psychosocial Support and Mental Health Services Helpline during the COVID-19 pandemic.

Methods: A secondary reflexive thematic analysis was conducted on 82 de-identified helpline call summaries (March 2020-October 2021). Data were analyzed using Braun and Clarke's six-phase analytic framework. Age-stratified, caller-type, and gender-differentiated analyses were used to examine demographic patterns within the dataset.

Results: The sample comprised predominantly male (89.0%) older adults with a mean age of 69.3 years (SD: 7.6). Approximately 52.4% (n=43) were proxy calls initiated by family members (primarily sons), while 47.6% (n=39) were direct calls from older adults. Six major themes emerged, including psychological distress and emotional vulnerability (39.0%, n=32), pandemic-related isolation (22.0%, n=18), health anxieties (26.8%, n=22), cognitive concerns (18.3%, n=15), vaccination-related concerns (22.0%, n=18), and family relationship and caregiver issues (17.1%, n=14). Older adults aged ≥80 years showed higher prevalence of isolation (36.4%, n=4) and cognitive concerns (36.4%, n=4). Direct callers more frequently reported psychological distress (46.2%, n=18), whereas proxy callers more often reported cognitive concerns (25.6%, n=11) and family relationship strain (25.6%, n=11).

Conclusions: Helpline interactions provided insight into the psychosocial concerns prompting older adults and their families to seek support during the COVID-19 pandemic. The findings highlight the importance of age-responsive, linguistically accessible, and family-inclusive mental health services during public health emergencies in low- and middle-income country contexts.

## Introduction

The COVID-19 pandemic generated unprecedented psychosocial stressors worldwide, with older adults experiencing a disproportionate burden of mental health challenges. Beyond the elevated risk of infection, older adults faced multiple compounding stressors, including loss of independence, disruption of social engagement due to lockdowns and social distancing measures, restricted access to healthcare, and heightened mortality-related anxiety [1-3]. In India, strict lockdown measures implemented beginning in March 2020 further intensified these vulnerabilities, resulting in profound isolation from family and community support systems, severely limited access to healthcare services, and increased fears of COVID-19 transmission [3].

India’s mental health infrastructure remains insufficient to meet population needs. According to the World Health Organization, India has approximately 0.3 psychiatrists, 0.07 psychiatric social workers, 0.07 clinical psychologists, and 0.10 psychiatric nurses per 100,000 people [4,5], contributing to substantial treatment gaps. Despite these structural constraints and growing international evidence documenting increased anxiety and depression among older adults during the COVID-19 pandemic [1,2,6], limited research has examined psychosocial help-seeking behavior through India’s national mental health service systems [2,6].

In response to the increasing psychosocial needs during the pandemic, the National Institute of Mental Health and Neurosciences (NIMHANS) launched the National Psychosocial Support and Mental Health Services Helpline on March 24, 2020. Operating across 13 Indian languages and staffed by more than 600 trained counselors from nodal mental health institutes, the helpline provided psychoeducation, stress management strategies, and referrals for psychiatric care [7]. By May 2021, the service had received approximately 340,980 calls and delivered over 53,685 psychosocial interventions.

This study aimed to identify the psychosocial factors prompting help-seeking among older adults and their family members who contacted the National Psychosocial Support and Mental Health Services Helpline during the COVID-19 pandemic. Examining these helpline interactions provides insight into the psychosocial concerns that motivated individuals to seek support during the acute pandemic phase. Understanding these patterns may inform culturally responsive mental health service delivery in low- and middle-income country contexts, particularly during public health emergencies.

To contextualize the findings, this analysis draws on several theoretical perspectives. The Stress-Coping Model suggests that psychological distress arises when environmental stressors exceed individuals’ perceived coping resources; during the pandemic, threats such as infection risk, healthcare disruption, social isolation, and economic uncertainty likely exceeded many older adults’ adaptive capacities [8]. Social Support Theory highlights the protective role of social relationships in buffering mental health deterioration; the high prevalence of proxy calls (52.4%) reflects the importance of family as a primary pathway for help-seeking [9]. The Life-Course Developmental Framework conceptualizes successful aging through selective optimization with compensation (SOC), mechanisms that were disrupted during the pandemic across older age groups [10]. The Health Belief Model explains health decision-making through perceptions of threat and beliefs about the effectiveness of preventive actions, which may be particularly relevant for vaccine-related help-seeking [11]. Finally, the Relational Resilience Framework conceptualizes families as relational systems that may function as both sources of support and stress, providing a useful lens for understanding caregiver-mediated help-seeking behaviors [12].

## Materials and methods

Study design and reporting standards

The study was conducted using data from the National Psychosocial Support and Mental Health Services Helpline operated by the NIMHANS, Bengaluru, Karnataka, India. This study employed a secondary reflexive thematic analysis of de-identified helpline call summaries following Braun and Clarke’s six-phase analytic framework [13]. Reporting was guided by the Standards for Reporting Qualitative Research (SRQR) to ensure transparency and methodological rigor.

Setting and data source

Data were obtained from the NIMHANS National Psychosocial Support and Mental Health Services Helpline [7], established on March 24, 2020, as India’s national psychosocial response to the COVID-19 pandemic. The helpline operated across 13 Indian languages and was staffed by over 600 trained counselors from multiple nodal mental health institutes. Incoming calls were routed through an interactive voice response system (IVRS) that categorized callers via keypress options to enable targeted routing. Call summaries were generated through systematic recording, transcription, and summarization procedures and documented the primary psychosocial concerns presented by callers.

Sampling strategy

Eligible records included calls made by or on behalf of older adults aged ≥60 years between March 2020 and October 2021 that contained substantive psychosocial content and were conducted in Hindi, Tamil, or English. Administrative-only inquiries, incomplete or corrupted recordings or summaries, and calls lacking psychosocial content were excluded. An a priori target sample of 80-100 summaries was determined based on published guidance regarding qualitative thematic saturation [14], resulting in a final analytic sample of 82 call summaries.

Data preparation

All personally identifiable information, including names, family member identifiers, counselor identities, contact details, and institutional affiliations, was removed prior to analysis. Researcher positionality was explicitly acknowledged, including the researcher’s institutional affiliation with NIMHANS and the potential for confirmation bias. Reflexive strategies were employed to examine pre-existing assumptions, including expectations that the pandemic would precipitate anxiety and depression, that helpline services would be perceived as helpful, and that findings might align with existing international evidence.

Data analysis

Systematic coding across all 82 call summaries identified initial codes: anxiety, depression, sleep disruption, isolation, lockdown restrictions, reduced social engagement, COVID-19 infection fear, health-related concerns, chronic physical health conditions, cognitive symptoms, grief and loss, vaccine concerns, family support, substance use, and financial stressors. These codes were grouped into candidate themes based on semantic and conceptual relationships. Thematic validity was evaluated through consistency re-coding, demographic variation analysis, and examination of negative cases, resulting in the finalization of six major themes with clearly defined semantic and conceptual boundaries.

Demographic analysis

Age-stratified analysis examined the distribution of themes across three age groups: 60-69 years (younger-old), 70-79 years (old), and ≥80 years (oldest-old). Caller-type differentiation compared direct callers and proxy callers across themes. Gender-based analyses were interpreted cautiously due to the pronounced gender imbalance within the sample (predominantly male callers).

Statistical analysis

Descriptive demographic distributions and theme frequencies were summarized using proportions. Chi-square tests (χ²) were used to examine associations between selected categorical demographic variables where appropriate. Statistical analyses were conducted using the IBM SPSS Statistics for Windows, Version 28 (Released 2021; IBM Corp., Armonk, New York, United States).

Trustworthiness

Analytic quality was evaluated using criteria of transparency, coherence, interpretive depth, and credibility. The six final themes captured the breadth of psychosocial concerns expressed by callers, while interpretive depth was supported through attention to contextual meaning beyond surface categorization. Data saturation was indicated by pattern stability by the 70th summary, after which no new codes emerged.

Ethical considerations

Ethical approval for the study was obtained from the NIMHANS Ethics Committee for Research (NIMH/A&E-SA3/Ph.D/PSSDM/ADC/2021-22). All data were de-identified in accordance with the Indian Council of Medical Research guidelines. The researcher disclosed institutional affiliation with the helpline and acknowledged potential sources of bias. No primary participant risks were identified.

## Results

Demographic characteristics

The analytic sample comprised 82 call summaries from older adults accessing the NIMHANS Helpline (March 2020-October 2021). The sample was predominantly male (n=73, 89.0%), with eight female callers (9.8%) and one caller of unspecified gender (1.2%). Mean age was 69.3 years (SD: 7.64, range: 60-90). Age distribution showed 57.3% in the younger-old category (60-69 years, n=47), 29.3% in the old category (70-79 years, n=24), and 13.4% in the oldest-old category (80+ years, n=11).

Notably, 52.4% of calls (n=43) were proxy calls initiated by family members, predominantly adult sons (55.6% of proxy calls), followed by daughters (7.3%), grandsons (27.0%), and granddaughters (4.3%). Direct calls from older adults themselves accounted for 47.6% (n=39). This near-equal distribution reflects mixed help-seeking patterns, where family-centred support is culturally normative in Indian contexts, yet significant proportions of older adults self-advocate. Table 1 presents the distribution of caller types across age groups.

**Table 1:** Caller type by age group Distribution of direct and proxy callers across age groups among older adults accessing the National Institute of Mental Health and Neurosciences (NIMHANS) National Psychosocial Support and Mental Health Services Helpline during the COVID-19 pandemic.

Age Group (years)	Direct Callers, n (%)	Proxy Callers, n (%)	Total, n (%)
60-69 (Younger-old)	26 (66.7)	21 (48.8)	47 (57.3)
70-79 (Old)	10 (25.6)	14 (32.6)	24 (29.3)
≥80 (Oldest-old)	3 (7.7)	8 (18.6)	11 (13.4)
Total	39 (100)	43 (100)	82 (100)

Age-stratified analysis by caller type

Proxy callers were slightly older (mean 70.3 years vs 68.2 years; Δ=2.1 years). Chi-square analysis showed no significant association between age group and caller type (χ²=3.10, p=0.212). Notably, 55.3% of younger-old (60-69 years) were direct callers, compared to 48.6% in the proxy group. Conversely, 72.7% of the oldest-old (80+) were proxy callers, versus 27.3% direct callers, consistent with age-related functional decline necessitating family mediation.

Gender distribution

No significant difference in gender distribution between direct and proxy caller groups (χ²=0.02, p=0.89). Female callers, though underrepresented, were concentrated in the 70-79 age range and showed a higher prevalence of proxy-mediated help-seeking (75% vs 50% for direct-calling females).

Six major psychosocial themes

Percentages represent the proportion of the total analytic sample (N=82). Sub-themes are not mutually exclusive, and individual calls may contribute to multiple thematic categories.

Theme 1: Psychological Distress and Emotional Vulnerability (39.0%, n=32)

The most prevalent theme encompassed anxiety, depressive symptoms, and sleep disruption-reflecting the pandemic's multifaceted emotional toll [1,15].

Illustrative excerpt: "A 60-year-old man reported persistent anxiety and depressive symptoms despite pharmacotherapy".

Sub-themes included: Anxiety as primary presenting concern (30.5%, n=25); sleep disruption linked to psychological distress (20.7%, n=17); depressive symptoms and anhedonia (17.1%, n=14).

Theme 2: Pandemic-Related Isolation and Loneliness (22.0%, n=18)

Enforced social distancing caused profound disruption to social engagement and led to a sense of identity erosion [16].

Illustrative excerpt: "An 84-year-old retired railway worker and artist described restlessness and emotional unease after pausing craftwork due to illness; he experienced profound longing for purposeful engagement despite adequate physical health and sleep."

Sub-themes: Enforced confinement and behavioral changes (12.2%, n=10); loss of social engagement and identity disruption (11.0%, n=9).

Theme 3: Health Anxieties and Disease Concerns (26.8%, n=22)

Multi-layered health concerns reflected the intersection of the novel COVID-19 threat with the burden of age-related chronic disease [17].

Illustrative excerpt: "A caller on behalf of his 73-year-old father, experiencing sleep disturbances due to nocturnal leg pain complicated by diabetes, hypertension, and chronic kidney disease, requested guidance on medication adherence, sleep hygiene optimisation, and physician consultation post-lockdown."

Sub-themes: COVID-19-specific infection and transmission fears (14.6%, n=12); chronic disease management under pandemic constraints (14.6%, n=12).

Theme 4: Cognitive Decline and Aging Vulnerabilities (18.3%, n=15)

This theme integrated age-normative cognitive changes with existential challenges of aging. Grief and bereavement occurred in pandemic contexts marked by isolation-related deaths and disrupted mourning rituals [18].

Illustrative excerpt: "A woman called on behalf of her 85-year-old grandfather exhibiting cognitive decline (restlessness, disorientation, insomnia, appetite loss); she requested interim guidance given immediate physician access limitations."

Sub-themes: Memory problems and cognitive disorientation (12.2%, n=10); grief, bereavement, and existential reflection (9.8%, n=8).

Theme 5: Vaccination-Related Concerns (22.0%, n=18)

Nearly one-quarter of calls involved vaccine-related questions: safety concerns, adverse reaction worries, and logistical barriers [19]. This phase-specific theme reflected shifting psychosocial priorities as vaccine rollout progressed.

Illustrative excerpt: "A caller sought safety information regarding COVID vaccination for an elderly person with a prior paralysis episode and residual mobility/memory impairment."

Sub-themes: Vaccine safety and efficacy questions (n=10); post-vaccination adverse reactions and complications (n=8).

Theme 6: Family Relationships and Caregiver Support (17.1%, n=14)

Family relationships emerged as fundamental contextual factors shaping older adult mental health. The 52.4% prevalence of proxy calls reflects culturally normative family-centred help-seeking [20].

Illustrative excerpt: "A caller sought help for his 75-year-old father who remained semi-conscious despite COVID-negative status, possibly from emotional trauma; the son provided single-handed caregiving despite family neglect."

Sub-themes:** **Family members as facilitators of help-seeking (12.2%, n=10); family conflict and relational strain (7.3%, n=6).

Table 2 presents the age-stratified distribution of psychosocial themes. Younger-old (60-69 years) demonstrated elevated psychological distress (40.4%) and vaccine engagement (29.8%), suggesting high anxiety vulnerability and vaccine decision-making engagement. Oldest-old (80+ years) showed markedly elevated isolation prevalence (36.4%), cognitive concerns (36.4%), and family relationship strain (36.4%), indicating advancing age increases isolation and family-system involvement.

**Table 2:** Age-stratified thematic variation Distribution of psychosocial themes across age groups among older adults accessing the National Institute of Mental Health and Neurosciences (NIMHANS) National Psychosocial Support and Mental Health Services Helpline during the COVID-19 pandemic. Theme 1: Psychological Distress and Emotional Vulnerability; Theme 2: Pandemic-Related Isolation and Loneliness; Theme 3: Health Anxieties and Disease Concerns; Theme 4: Cognitive Decline and Aging Vulnerabilities; Theme 5: Vaccination-Related Concerns; Theme 6: Family Relationships and Caregiver Support.

Theme	60-69 years (n=47)	70-79 years (n=24)	80+ years (n=11)	Total (n=82)
Theme 1	19 (40.4%)	9 (37.5%)	4 (36.4%)	32 (39.0%)
Theme 2	8 (17.0%)	6 (25.0%)	4 (36.4%)	18 (22.0%)
Theme 3	13 (27.7%)	7 (29.2%)	2 (18.2%)	22 (26.8%)
Theme 4	5 (10.6%)	6 (25.0%)	4 (36.4%)	15 (18.3%)
Theme 5	14 (29.8%)	3 (12.5%)	1 (9.1%)	18 (22.0%)
Theme 6	6 (12.8%)	4 (16.7%)	4 (36.4%)	14 (17.1%)

Table 3 presents caller-type differentiation across psychosocial themes. Direct callers showed elevated psychological distress (46.2% vs 32.6%), indicating self-advocates were primarily emotionally distressed. Proxy callers demonstrated a higher prevalence of cognitive concerns (25.6% vs 10.3%) and family relationship strain (25.6% vs 7.7%), suggesting that family-initiated calls were primarily motivated by mental deterioration and family dynamics rather than older adult emotional distress.

**Table 3:** Caller-type differentiation Distribution of psychosocial themes among direct callers and proxy callers accessing the National Institute of Mental Health and Neurosciences (NIMHANS) National Psychosocial Support and Mental Health Services Helpline during the COVID-19 pandemic. Theme 1: Psychological Distress and Emotional Vulnerability; Theme 2: Pandemic-Related Isolation and Loneliness; Theme 3: Health Anxieties and Disease Concerns; Theme 4: Cognitive Decline and Aging Vulnerabilities; Theme 5: Vaccination-Related Concerns; Theme 6: Family Relationships and Caregiver Support.

Theme	Direct Callers (n=39)	Proxy Callers (n=43)	Total (n=82)
Theme 1	18 (46.2%)	14 (32.6%)	32 (39.0%)
Theme 2	7 (17.9%)	11 (25.6%)	18 (22.0%)
Theme 3	10 (25.6%)	12 (27.9%)	22 (26.8%)
Theme 4	4 (10.3%)	11 (25.6%)	15 (18.3%)
Theme 5	11 (28.2%)	7 (16.3%)	18 (22.0%)
Theme 6	3 (7.7%)	11 (25.6%)	14 (17.1%)

Gender differentiation

Due to severe gender imbalance (89% male, 9.8% female), robust gender-stratified analysis is not defensible. However, preliminary observations showed that female callers had a higher prevalence of Theme 4 (37.5% vs 17.1% in males) and Theme 6 (25.0% vs 15.8% in males). Male callers demonstrated a higher prevalence of Theme 5 (26.0% vs 12.5% in females). These patterns require confirmation in gender-balanced datasets.

## Discussion

Psychological distress and emotional vulnerability

Psychological distress was the most prevalent theme (39.0%), reflecting the cumulative impact of the pandemic on emotional well-being [1,15,21]. Notably, older adults rarely framed distress as a clinical illness; instead, they described it as reasonable responses to ongoing stressors, such as infection risk, news exposure, and loss of control [20,22]. This pattern aligns with qualitative evidence showing older adults contextualize psychological distress within daily life narratives rather than medical models [22]. The helpline's low-threshold accessibility allowed callers to express suffering without formal mental illness identification, a vital service function in contexts with mental health stigma [23].

Pandemic-related isolation and loneliness

Isolation and loneliness (22.0% overall; 36.4% in the oldest-old) represented profound pandemic consequences. Importantly, older adults' experiences of isolation reflected not merely the absence of social contact but the erosion of meaningful roles and reciprocal relationships [20,22]. The helpline provided temporary relational continuity, consistent with evidence that empathetic telephonic interaction alleviates perceived isolation [24]. The elevated isolation prevalence in the oldest-old (36.4%) may reflect compounded vulnerability to lockdown restrictions, reduced mobility, and family disruption.

Health anxieties and disease concerns

Health anxieties (26.8%) reflected realistic intersections between the novel COVID-19 threat and the chronic disease burden. Unlike exaggerated or irrational fears, older adults' health concerns were grounded in legitimate risk assessments and genuine barriers to healthcare access. The helpline's provision of reassurance, clarity, and referral pathways addressed a critical need: uncertainty regarding symptom interpretation and healthcare availability significantly contributes to psychological distress in older adulthood [17,24].

Cognitive decline and aging vulnerabilities

Cognitive concerns (18.3% overall; 36.4% in the oldest-old) were often reported by proxy callers, illustrating the family's role in recognizing cognitive deterioration. Pandemic stress and isolation can exacerbate cognitive symptoms in vulnerable older adults [6,17]. Notably, limitations of telephonic assessment (the absence of visual cues and standardized cognitive testing) underscore the necessity of careful escalation and referral protocols rather than premature diagnostic inference [23,24].

Vaccination-related concerns

Vaccination concerns (22.0% overall; 29.8% in the younger-old) reflected phase-specific psychosocial changes as the vaccine rollout progressed. Older adults sought personalized, context-specific information that accounted for comorbidities and prior experiences [19,21]. The helpline functioned as a trusted information source; research indicates that clear, trustworthy information decreases anxiety, while media contradictions increase fear [21,25]. The lower vaccine concern prevalence in the oldest-old (9.1%) may reflect lower digital/media engagement or family information gatekeeping.

Family relationships and caregiver support

The 52.4% prevalence of proxy calls is consistent with culturally normative family-centered help-seeking in Indian contexts. Family-mediated calls illustrated caregiver strain, emotional burden, and relational tension [2,20,26,27]. Proxy callers, predominantly adult sons, served as critical intermediaries between older adults and healthcare systems, roles that intensified amid pandemic service disruptions. Recognizing caregivers as secondary service users is essential for developing geriatric mental health responses [20,22].

Clinical and service implications

Accessibility and Low-Threshold Entry

Telephone helplines provide accessible, low-barrier pathways, particularly valuable in resource-limited contexts where in-person services are disrupted or unavailable [24,28].

Counselor Training

Counselors require training in recognizing older adults' indirect emotional expressions, such as somatic concerns, sleep disturbances, and contextual narratives, rather than relying on psychological terminology, to provide validating responses without premature diagnostic framing [20,22].

Healthcare Integration

Mental health helplines require integration with primary and geriatric care systems to address health-related anxiety by establishing clear referral pathways and updating service information, thereby mitigating care continuity gaps [17,24].

Cognitive Concern Protocols

Telephonic cognitive assessment limitations necessitate protocols that emphasize interim guidance, caregiver involvement, and timely in-person referral rather than overinterpretation of symptoms arising during acute stress or isolation [17,23,24,29].

Family-Inclusive Interventions

Given elevated caregiver burden, services should extend validation, guidance, and support to family members alongside older adults, acknowledging caregivers' roles as help-seeking facilitators and as individuals experiencing significant psychological burden [20].

Vaccine-Related Communication

Personalized, context-specific vaccine information that accounts for comorbidities and prior experiences reduces anxiety and supports informed decision-making, particularly when media exposure is distressing [21,25].

Limitations

This study draws on helpline data reflecting spontaneous help-seeking during a public health emergency; however, helpline users are self-selected, and older adults without telecommunication access remain underrepresented; therefore, the findings should be interpreted as contextual insights into help-seeking experiences rather than statistically generalizable estimates [23,24]. Reliance on telephonic summaries constrains assessment of cognitive and affective states; distress is narrated in moments of need rather than clinically assessed. Severe gender imbalance (89% male) limits robust gender-stratified analysis; findings may not generalize to older female adults. Themes are shaped by helpline interactional context and the researcher's interpretive positioning; however, this positioning is transparent and acknowledged [22]. Data capture a 13-month pandemic window and may not reflect long-term psychosocial patterns.

## Conclusions

This secondary analysis of 82 helpline calls reveals the nature and patterns of psychosocial distress motivating older adults and families to seek help during COVID-19's acute phase in India. The sample's predominantly male (89%) composition and near-equal distribution of direct/proxy calls (47.6%/52.4%) reflect India's demographics and help-seeking patterns. Six psychosocial themes: psychological distress, isolation, health anxieties, cognitive concerns, vaccine-related questions, and family dynamics captured the breadth of callers' psychosocial landscape.

Age-stratified findings suggest developmental shifts: younger-olds prioritize psychological distress and vaccine information; oldest-olds face disproportionate isolation, cognitive concerns, and family involvement. Caller-type differentiation shows direct callers experience greater emotional distress, while proxy callers seek help primarily regarding cognitive and family problems, suggesting complementary help-seeking pathways.

The helpline's 13-language, geographically dispersed infrastructure and trained counselor network represented an innovative service model for culturally responsive, accessible mental health support. The findings highlight telephone-based helplines as potentially viable and acceptable services for older adults in low- and middle-income country contexts, particularly when linguistically inclusive and empathetically delivered. Future research should examine longer-term psychosocial trajectories, the linkages between interventions and outcomes, and gender-specific patterns in larger, gender-balanced samples.

These findings highlight the importance of accessible, family-inclusive, and age-responsive mental health services as key components of public health emergency preparedness for aging populations, emphasizing accessibility, family-inclusive approaches, and age-responsive strategies as essential components of public health emergency response for aging populations.
